# Harmonic motion imaging for pancreatic tumor detection and high-intensity focused ultrasound ablation monitoring

**DOI:** 10.1186/2050-5736-3-S1-O81

**Published:** 2015-06-30

**Authors:** Hong Chen, Thomas Payen, Yang Han, Carmine Palermo, Kenneth Olive, Elisa Konofagou

**Affiliations:** 1 Columbia University, New York, New York, United States

## Background/introduction

Harmonic motion imaging (HMI) is a radiation force-based elasticity imaging technique that estimates tissue harmonic displacements induced by an oscillatory ultrasonic radiation force to assess tissue stiffness. The objective of this study was to evaluate the feasibility of applying HMI on pancreatic tumor detection and high-intensity focused ultrasound (HIFU) treatment monitoring.

## Methods

A transgenic mouse model of pancreatic cancer (KPC) as well as wild-type mice were used in this study. The HMI system consisted of a focused ultrasound transducer (FUS), which generated oscillatory radiation force that then induced harmonic tissue motion at 50 Hz at the focus, and a diagnostic ultrasound transducer, which detected the axial tissue displacement within the targeted region using 1D cross-correlation of acquired radiofrequency signals. For pancreatic tumor detection, HMI displacement maps were generated for pancreatic tumors in transgenic mice and healthy pancreases of wild-type mice. For pancreatic tumor ablation monitoring, FUS was used to induce thermal ablation and tissue motion at the same time, allowing HMI monitoring without interrupting tumor ablation. HMI images were acquired at 3-s intervals to monitor changes in tissue stiffness during ablation. All pancreases were excised immediately after sonication for histological evaluation, including hematoxylin and eosin (H&E) staining, cleaved caspace-3 antibody staining, and trichrome staining.

## Results and conclusions

The obtained HMI displacement maps showed a high contrast between normal and malignant tissues (with an average lesion-to-normal displacement ratio of 2.4). HMI monitoring of the HIFU ablation depicted consistent pancreatic stiffening with a mean HMI displacement reduction rate of 25% after 2 min ablation, and the formation of thermal lesions was confirmed by the histological analysis. H&E staining confirmed accurate targeting of the pancreatic tumor. Cleaved caspace-3 antibody staining confirmed apoptosis induced by HIFU ablation. Trichrome staining revealed the damaging effects of HIFU on the stromal matrix. This study demonstrated thus for the first time the feasibility of HMI in pancreatic tumor detection and HIFU ablation monitoring. It was also the first application of a radiation-force based technique for HIFU ablation monitoring of an abdominal organ.

**Figure 1:**
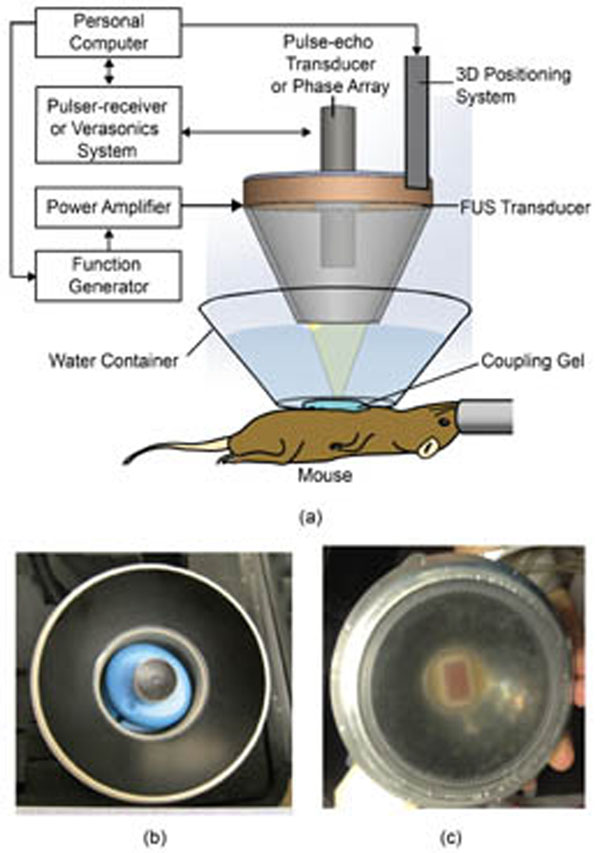
(a) Illustration of the experimental setup. (b) Picture of the 1D HMI transducers. (c) Picture of the 2D HMI transducers.

**Figure 2:**
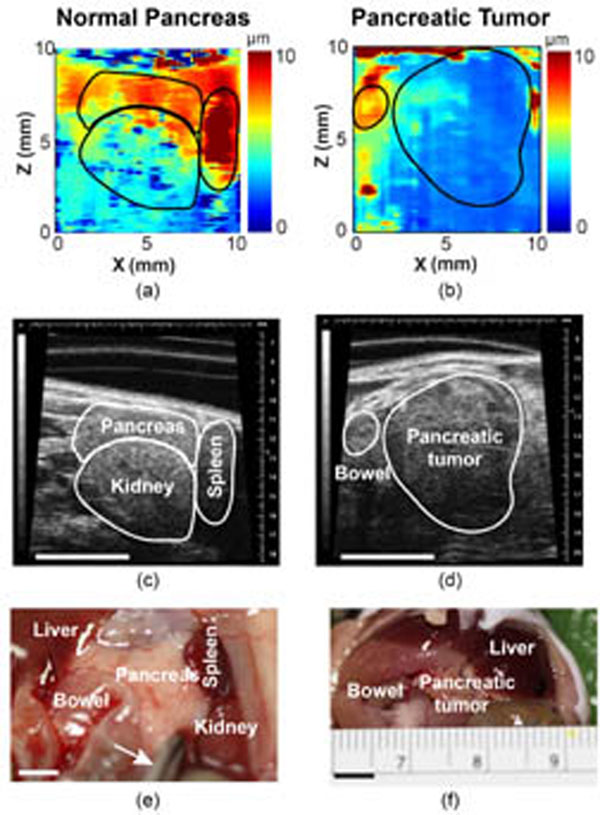
HMI displacement maps of normal pancreas and pancreas tumor. The corresponding Vevo B-mode images obtained at the same scanning plane are shown in (c) and (d), respectively. Pancreas locations were confirmed with biopsy shown in (e) and (f).

**Figure 3:**
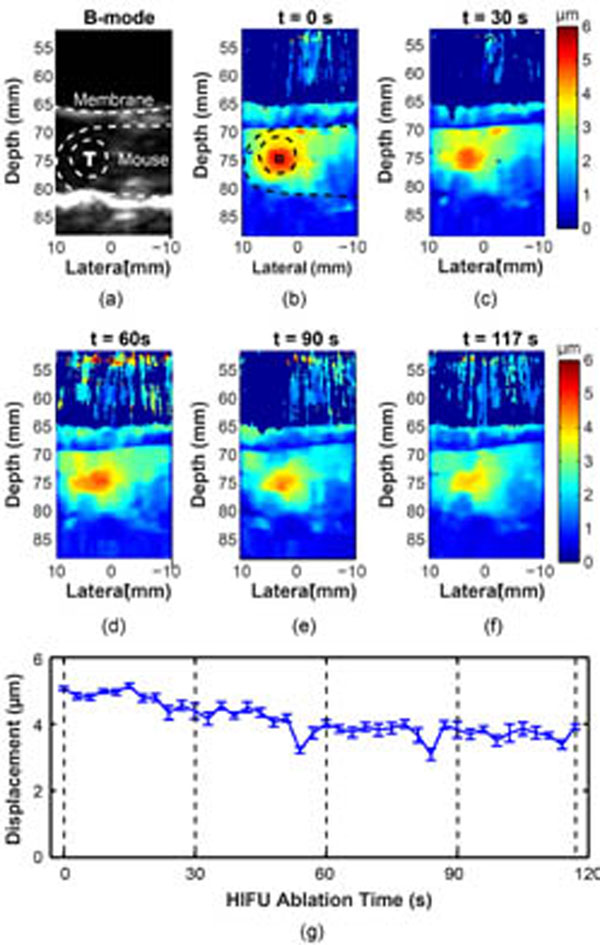
HMI displacement image at different HIFU treatment time point (b-f). The corresponding B-mode image obtained before HIFU ablation is shown in (a). (g) Mean and standard deviation of HMI displacements within the focal region (square in (a)) over time.

**Figure 4:**
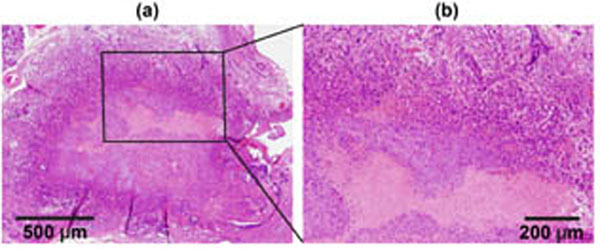
H&E staining images of the pancreatic tumor after HIFU ablation.

